# New Insights into the Pathogenesis and Treatment of Urinary Tract Infections

**DOI:** 10.3390/pathogens12101213

**Published:** 2023-10-03

**Authors:** Carmelo Biondo

**Affiliations:** Department of Human Pathology, University of Messina, Via C. Valeria n.1, 98125 Messina, Italy; cbiondo@unime.it

About 150 million people around the world experience urinary tract infections (UTI) every year, with adult women 30 times more likely to develop a UTI than men [1]. Most UTIs are still successfully treated with oral antibiotics, even though it has long been known that systemic antibiotic treatment accelerates the selection of antibiotic-resistant strains not only to the prescribed antibiotic but also to other antimicrobial agents [2]. The problem of antimicrobial resistance has been identified by the World Health Organization as one of the top 10 global public health threats [2]. In complex cases of UTI, to avoid the emergence of AMR, alongside the development of new non-antibiotic alternatives, there is a growing need to better target antibiotic therapies [3]. A recent review by Morris and colleagues [4] emphasizes the fact that the optimal treatment of UTI involves not only determining the antibiotic susceptibility profile of the pathogen, but also assessing the pharmacokinetic–pharmacodynamic (PK-PD) properties of the chosen agent, i.e., the likelihood that the chosen antibiotic can be present in the target tissue in sufficient concentrations and for sufficient time to resolve the infection. The authors therefore review the evidence for the use of direct instillation of antibiotics in the urinary tract [4]. The development of an intravesical antibiotic delivery route would not only avoid AMR, but also, by virtue of the negligible urothelial permeability of the drugs, safeguard non-urothelial microbial communities, including those residing in the gut that are critical to overall health. In their review, Morris et al. also highlight the fact that while an intravesical approach to drug delivery is promising and has attracted the interest of clinicians, a better delivery system needs to be developed, as drug delivery through standard drainage catheters carries a high risk of infection [4]. While several groups have investigated the use of functionalized antimicrobial particles suitable for intravesical treatment of UTI, and some have attempted to improve antibiotic instillation using nano-sized delivery platforms, none of these approaches have been approved for this use [5,6,7]. However, in light of the evidence supporting the use of the intravesical route of antibiotic administration, as outlined by authors in this review, there is a significant need for investment in novel technologies to enable controlled and targeted delivery of antibiotics.

Although UTIs are not usually a serious type of infection, they are a common health problem that affects millions of people each year and are usually treated empirically [8]. In both community and hospital settings, for both uncomplicated (uUTI) and complicated (cUTI) cases, the bacterial species most commonly associated with UTI are *E. coli*, *Klebsiella* spp. and *Proteus mirabilis* among Gram-negative bacteria, and *Enterococcus faecalis* and *Staphylococcus* spp. among Gram-positive bacteria [9]. The treatment of UTIs is becoming increasingly complex due to the rise of multi-drug-resistant strains, leading to significant antibiotic resistance and economic implications associated with the treatment of these infections [10]. To prevent the emergence of these phenomena, the implementation of effective antimicrobial stewardship is essential. A recent review by Mancuso et al. outlines the key interventions needed to control the spread of multi-drug-resistant isolates in UTI [9]. The challenges identified are to discourage the treatment of asymptomatic bacteriuria, to reduce the use of broad-spectrum fluoroquinolones, and to combat the development of resistance by adhering to the recommended dosages and courses of treatment [9]. What are the primary pathogenic mechanisms of bacteria causing UTI? What are the latest advancements in alternative therapies, besides antibiotics, for controlling the spread of multi-drug-resistant isolates that are responsible for UTIs? For both questions, the review by Mancuso et al. comprehensively examines the mechanisms of antimicrobial pathogenicity of the major UTI pathogens for which new antimicrobial agents are urgently needed, as well as available or novel non-antibiotic approaches for the treatment of these pathogens [9].

*Proteus mirabilis* (PM) is a Gram-negative, rod-shaped bacterium known for its swarming motility and urease activity [11]. This bacterium is the most common cause of urinary tract infections associated with catheter use [11]. The urease catalyzes the hydrolysis of urea to produce ammonia (NH3) and carbon dioxide (CO_2_). This process raises the pH level of urine which leads to salt precipitation and crystal formation. The crystals then deposit on the catheter, resulting in the formation of struvite stones [12]. In some cases, serious complications such as septicemia and endotoxic shock may occur, potentially proving fatal [13]. Although various virulence factors of *P. mirabilis* related to different infection stages have been described, the exact function of specific cell surface components concerning the adhesion and invasion of uroepithelial cells remains unclear [14,15,16]. In their original publication, Herout et al. examine the ways in which bacterial surface components affect PM pathogenicity, with a focus on the adherence of indwelling devices that lead to biofilm establishment [17]. The study also investigates uroepithelial cell attachment and invasion after biofilm formation. In order to assess the importance of specific cell surface components in the pathogenicity of *Proteus mirabilis*, the researchers utilize transposon and targetron mutants of genes that had been previously generated [17]. Their research provides a comprehensive view of bacterial surface structures that are critical for PM adhesion and invasion of bladder epithelial cells [17]. The identification of these critical components is an important first step in the development of targeted therapies that can interfere with key stages of *Proteus mirabilis* pathogenesis, thereby halting the associated morbidity and mortality and preventing further negative health outcomes.
